# Prolonged Soil Frost Affects Hydraulics and Phenology of Apple Trees

**DOI:** 10.3389/fpls.2016.00867

**Published:** 2016-06-20

**Authors:** Barbara Beikircher, Claudia Mittmann, Stefan Mayr

**Affiliations:** Institute of Botany, University of InnsbruckInnsbruck, Austria

**Keywords:** fine roots, native embolism, refilling, starch, tree hydraulics

## Abstract

Restoration of an adequate water supply in spring is a prerequisite for survival of angiosperm trees in temperate regions. Trees must re-establish access to soil water and recover xylem functionality. We thus hypothesized that prolonged soil frost impairs recovery and affects hydraulics and phenology of *Malus domestica* var. ‘Golden Delicious.’ To test this hypothesis, over two consecutive winters the soil around some trees was insulated to prolong soil frosting, From mid-winter to early summer, the level of native embolism, the water and starch contents of wood, bark and buds were quantified at regular intervals and findings correlated with various phenological parameters, xylogenesis and fine root growth. The findings confirm that prolonged soil frost affects tree hydraulics and phenology but the severity of the effect depends on the climatic conditions. In both study years, percentage loss of hydraulic conductivity (PLC) decreased from about 70% at the end of winter to about 10% in May. Thereby, xylem refilling strongly coincided with a decrease of starch in wood and bark. Also treated trees were able to restore their hydraulic system by May but, in the warm spring of 2012, xylem refilling, the increases in water content and starch depolymerization were delayed. In contrast, in the cold spring of 2013 only small differences between control and treated trees were observed. Prolongation of soil frost also led to a delay in phenology, xylogenesis, and fine root growth. We conclude that reduced water uptake from frozen or cold soils impairs refilling and thus negatively impacts tree hydraulics and growth of apple trees in spring. Under unfavorable circumstances, this may cause severe winter damage or even dieback.

## Introduction

Freezing winter conditions in some temperate, continental climates can severely impair water transport in the xylem by freeze-thaw induced embolism. Impaired water transport affects tree water status, water uptake from the soil and especially water supply of the leaves. The prompt restoration of a tree’s water-transport system in spring is thus a prerequisite for growth and fruiting that season (Sperry et al., 1994; Ameglio et al., 2002). Water transport in the xylem is driven by a pressure gradient and requires that the water columns are hydraulically continuous (Sperry et al., 1988a; Tyree and Zimmermann, 2002; Choat et al., 2012). Drought and/or freeze-thaw events can break these columns (cavitation) resulting in air-filled conduits (embolisms; Tyree and Zimmermann, 2002). Air is soluble in water but not in ice. Thus, freezing of xylem water leads to the formation of air bubbles (Hammel, 1967). Depending on the size of the bubbles and the xylem tension (negative pressure), these bubbles can expand during thawing and cause air embolisms (Sperry and Sullivan, 1992; Tyree et al., 1994; Hacke and Sauter, 1996; Cochard et al., 2001; Mayr et al., 2007; Sevanto et al., 2012). A critical factor thereby is conduit diameter, as it is determines the radius of curvature of the approximately hemispherical menisci at the two ends of a bubble (Sperry and Sullivan, 1992; Pittermann and Sperry, 2006; Choat et al., 2011). Accordingly, angiosperms are generally more prone to freeze-thaw-induced embolisms than gymnosperms (e.g., Hacke et al., 2001; Sperry et al., 2006). In addition, winter embolisms can also be induced by drought. When xylem tensions exceed species-specific thresholds, air can be sucked into water-filled conduits from the air-filled intercellular spaces or adjacent embolized conduits (e.g., Sperry et al., 1988a; Tyree and Zimmermann, 2002; Christensen-Dalsgaard and Tyree, 2014). In contrast to freeze-thaw induced cavitation, relationships between xylem anatomy and drought-induced cavitation are far more complex with pit membrane properties playing a major role (Hacke et al., 2001; Wheeler et al., 2005; Sperry et al., 2006; Cai and Tyree, 2010).

Depending on xylem structure, winter xylem tensions and the number of freeze-thaw cycles, many tree species suffer an almost complete loss of hydraulic conductance in winter (Sperry et al., 1988b, 1994; Cochard and Tyree, 1990; Sperry and Sullivan, 1992; Hacke and Sauter, 1996; Cochard et al., 2001; Jaquish and Ewers, 2001; Ameglio et al., 2002; Christensen-Dalsgaard and Tyree, 2014). This may not be particularly damaging, however, as full water-transport capacity is not necessarily required during winter with low transpiration rates and reduced or blocked water uptake from the soil in many deciduous angiosperms. Thus, embolisms do not generally pose special problems for these deciduous plants in winter. However, circumstances quickly become critical in spring when water demand increases due to increased evaporation rates, on-setting transpiration and growth processes, (e.g., Sperry et al., 1994; Ameglio et al., 2002; Hao et al., 2013). Therefore, tree species that are unable to avoid winter embolism, must have mechanisms that allow rapid restoration of functionality of their water transport systems in order to avoid growth limitation or dieback through shoot desiccation.

Recovery of hydraulic conductance in spring can occur through the refilling of embolized xylem conduits and/or through the differentiation of new ones (e.g., Sperry and Sullivan, 1992; Sperry et al., 1994; Cochard et al., 2001; Jaquish and Ewers, 2001; Ameglio et al., 2002; Christensen-Dalsgaard and Tyree, 2014; Mayr et al., 2014). It is known that many diffuse-porous trees are able to refill some of their embolized conduits in spring (Sperry et al., 1988b, 1994; Sperry and Sullivan, 1992; Hacke and Sauter, 1996; Cochard et al., 2001; Christensen-Dalsgaard and Tyree, 2014). Although such refilling has been demonstrated for many species (see Brodersen and McElrone, 2013, for a detailed review), the mechanisms involved are still a matter of debate. Thermodynamic principles suggest that the refilling of an embolized xylem conduit should be possible only when xylem water pressure is near to or above zero, relative to the atmosphere (Yang and Tyree, 1992; Cochard et al., 2001; Nardini et al., 2011; Delzon and Cochard, 2014). Several angiosperm genera are able to generate positive xylem pressures in the root or stem prior to leaf flushing, and these would be sufficient to dissolve embolisms (Sperry et al., 1987, 1994; Cochard et al., 2001; Ewers et al., 2001; Ameglio et al., 2002; Brodersen and McElrone, 2013; Hao et al., 2013). However, there also seem to be many species which are able to refill embolized conduits in the absence of positive root or stem pressures (Tyree et al., 1999; Cochard et al., 2001; Salleo et al., 2006; Nardini et al., 2011; Brodersen and McElrone, 2013). Several studies suggest that this may be related to starch depolymerisation in xylem parenchymal cells (Salleo et al., 2004; Zwieniecki and Holbrook, 2009; Nardini et al., 2011; Secchi and Zwieniecki, 2012). These cells may also provide some of the water required for the refilling. This idea is supported by the observation of droplets in the refilling grapevine conduits visualized using high-resolution x-ray computer tomography by Brodersen et al. (2010). However, if the xylem is highly embolized, the amount of available water in the xylem parenchyma cells may not be sufficient. Observations of alterations in carbohydrate metabolism and related enzyme activities as well as the up-regulating of aquaporin genes responsible for the water movement from phloem to xylem support the hypothesis of phloem as second source for sugar and water (Salleo et al., 2006; Zwieniecki and Holbrook, 2009; Nardini et al., 2011; Secchi and Zwieniecki, 2011, 2012; Brodersen and McElrone, 2013).

However, water stored in xylem and phloem can only be the first source of water for refilling. In spring, thus sufficient water supply from the soil is not only essential for imminent transpiration and growth but also for xylem refilling. Frozen soils and stem bases prevent water uptake and flow, respectively, and can lead to severe water stress when water is lost by stomatal and/or peridermal transpiration (Larcher, 2003; Beikircher and Mayr, 2013). Water uptake from the soil, though, is also impaired at positive but low soil temperatures. The prime reason therefore is the strong increase in water flow resistance caused by structural changes in the biomembranes of roots which change from a liquid-crystal to a solid-gel state leading to a decrease in cell permeability (Grossnickle, 1988; Ameglio et al., 1990). Additionally, also the increased transfer resistance between soil and root due to a higher viscosity of water at low temperatures may play a role (Larcher, 2003; Pallardy, 2008). Last but not least, the growth of roots is strongly reduced at low soil temperatures, whereby the critical temperature limits vary among species and habitats (Kozlowski et al., 1991; Larcher, 2003). As it is assumed that water uptake occurs mainly over fine roots (Pallardy, 2008), inhibited fine root growth in spring may thus negatively affect embolism repair.

Several studies have shown that delayed soil thawing at contemporaneously high air temperatures negatively affect water relations in terms of sapflow and lead to severe or even lethal damage in conifers of boreal regions: water shortage by delayed or inhibited water uptake can lead to xylem cavitation and in consequence to reduced potential efficiency of Photosystem II and foliar injuries (e.g., Repo et al., 2005, 2008). However, to our knowledge there are no studies dealing with xylem functionality and spring recovery under prolonged soil frost in angiosperm species. In apple orchards in northern Italy, so called ‘winter damage’ occurs at irregular intervals. Symptoms show in early spring and take the form of delayed leaf-flushing, dieback of parts of the crown or even of whole trees. Most affected by this phenomenon are young apple trees having reduced water storage capacity and growing on sites with delayed soil warming (north-exposed, shaded). Based on climatic conditions in the study area as well as measurements on frost hardiness from autumn to spring (Pramsohler et al., 2012; Pramsohler and Neuner, 2013) on trees of the present study, frost damage of buds and branches can be excluded. We hypothesized that prolonged soil frost in spring delays the recovery of the hydraulic system and thus negatively impact tree growth. To test this idea, soil frosting was artificially prolonged by insulating the soil under selected apple trees in two successive years. From mid-winter to early summer, the level of embolism, the water contents of wood, bark and buds, and the starch contents of wood and bark were measured at intervals in treated and control trees. Tree phenology was also determined including bud phenology, current year ring width and differences in fine root growth.

## Materials and Methods

### Plant Material, Climate, and Experimental Design

The field experiment was carried out in a commercial orchard in northern Italy (Tarsch, South Tyrol, 850 m a.s.l.; 46°37′N, 10°52′NE) on the apple cultivar *Malus domestica* var. ‘Golden Delicious’ trees [about 13 years old, 3 m tall, mean diameter at breast height (DBH) 64 mm].

**FIGURE 1:**
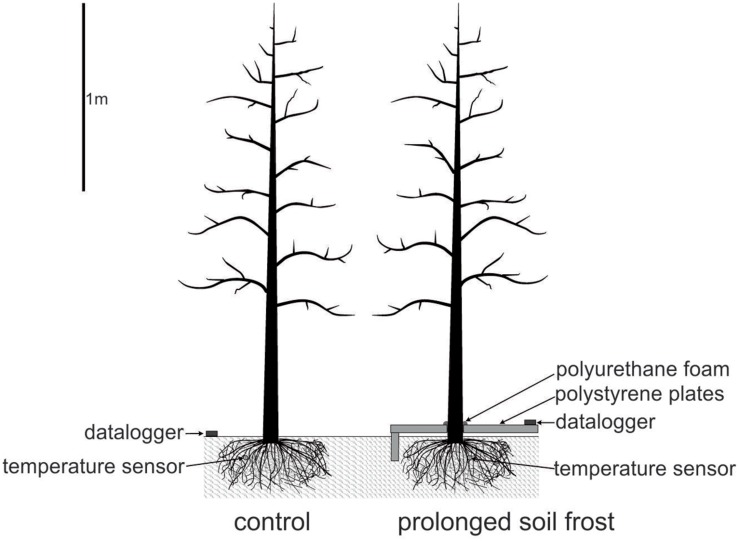
**Field experiment to simulate prolonged soil frost.** Soil around treated trees was covered with Styrofoam insulation sheets and gaps filled with polyurethane foam. Soil temperature at 10 cm depth was measured with a sensor connected to a data logger.

The orchard is situated in an inner alpine dry valley with exceptionally high sunshine duration (315 days), high annual mean temperature (9.6°C) and low precipitation (450–550 mm). The field treatments were imposed from February 2012 (about mid-winter) to March 2012 (spring), and repeated on eight other trees in the adjacent tree row from February 2013 to April 2013. In both years, the frozen soil around eight trees was insulated with Styrofoam roof-insulation sheets (Roofmate^TM^, 40 mm × 600 mm × 1250 mm) to prolong soil frosting. The sheets were mounted along the row over an area of 5 m × 0.6 m. Along the margins of the insulated area, vertical stripes of insulation sheets were inserted to a depth of 0.15 m to reduce lateral heat inflow from the surrounding un-insulated areas (see **Figure 1**). Gaps around stems and between sheets were filled with polyurethane foam (Soudafoam All Seasons^TM^, Soudal). For control trees, eight un-insulated trees in the respective adjacent row were chosen. This experimental design may lead to masked responses if investigated parameters are highly variable within the orchard. However, in the framework of another study trees were chosen randomly over the same orchard and no significant differences in water relation and phenology between trees observed (Beikircher, unpublished).

Air temperature and relative humidity (sensor EMS 33) at 2.5 m (in the upper crown) and soil temperatures (sensor Pt 100) at 10 cm depth (the main rooting depth) of both plots were measured at 1 min intervals and the 15 min means were accumulated in a data logger (ModuLog 3029; sensors and data logger of Environmental Measuring System, Brno, Czech Republic). In 2013, soil water potential in 10 cm depth was measured with two gypsum block sensors connected to a data logger (MicroLog SP, EMS, Brno, Czech Republic). The insulation sheets were removed as soon as the soil temperatures beneath them rose above freezing. From January to July of each year, embolisms and the water contents of bark, wood, and buds and the starch contents of bark and wood were measured at regular intervals (about every 14–21 days) and shoot phenology was monitored. In April 2013, the length of white roots was analyzed immediately after the removal of the insulation.

### Sampling and Preparation of Branches

At each sampling date, five west-exposed branches (several years old) were chosen randomly out of the eight trees per treatment. Because of earlier pruning management, branches were highly branched and crooked and contained several long and short shoots. Branches were cut at the base, immediately re-cut under water above the first annual shoot and left in water under a dark plastic bag for 30–45 min. This procedure was of particular importance to avoid entrance of air at the cut surface and thus artificial embolism in (I) winter, when frozen branches were thawing and (II) after bud break due to transpiration. Branches were then wrapped in dark plastic bags and transported to the laboratory. There, three samples per branch were cut under water from shoots developed in the preceding growing season (i.e., 2012 and 2013, respectively) for analyses of native embolism, water, and starch content. In a precedent study (Beikircher and Mayr, 2015), this harvesting and sampling protocol was proved to be appropriate to preserve the hydraulic state in the shoots used for measurements by avoiding artifactual embolization (Wheeler et al., 2013) as well as refilling (Trifilo et al., 2014).

### Native Embolism, Water Content, and Midday Water Potential

Xylem recovery was assessed by measuring the level of native embolism from winter to spring. Samples about 40 mm long were de-barked, ends were re-cut several times with a sharp wood-carving knife and sealed under water in tubes connected to a “Xylem” hydraulic conductance and embolism measurement system (Bronkhorst, Montigny les Cormeilles, France). The level of native embolism was expressed as the percentage loss of hydraulic conductance (PLC) and measured as the hydraulic conductance at 4.5 kPa before (*k*_i_) and after (*k*_max_) removal of embolism by repeated high pressure flushes at 95 kPa for 20 min (Eq. 1):

$$PLC=100-\left(k_{i}/k_{\max}\times 100\right)$$

For measurements, distilled, filtered (0.2 μm) and degassed water was used, containing 0.005% (v/v) ‘Micropur Forte MF 1000F’ (a mixture containing Ag+ and sodium hypochlorite for water sterilization and preservation; Katadyn Products Inc., Wallisellen, Switzerland) to prevent microbial growth (Sperry et al., 1988a; Beikircher and Mayr, 2008).

For water content analyses, about 100 mm long samples were de-barked and the fresh weights (FW) of bark, wood, and terminal buds were measured with an analytical balance (Sartorius BP61S, 0.0001 g precision, Sartorius AG, Göttingen, Germany). After oven drying at 80°C for 48 h, dry weight (DW) was determined and the water content expressed as a percentage of DW (WC_%DW_) as:

$${WC}_{\%DW}=(FW-DW)/DW\times 100$$

From bud break to June, midday water potential were measured on selected days between 11:00 and 12:00 CET. Measurements were made on at least five end twigs per treatment and date. Prior to bud break, measurement of xylem water potential was not possible due to the small portion of living tissues.

### Starch Contents of Wood and Bark

For starch analyses, the bark was removed from samples (∼40 mm long) and cut into thin strips with scissors. The wood was sliced with a microtome (Sledge Microtome G.S.L. 1, Schenkung Dapples, Zurich, Switzerland). After oven drying to constant weight at 80°C for 48 h, samples were finely ground with a micro-dismembrator (Braun Biotech, Melsungen, Germany). The materials thus obtained were incubated twice in 80% ethanol (v/v) at 75°C, with polyvinylpyrrolidone (PVP 40, Sigma Chemicals, USA) being added to bind polyphenols during the first incubation. The supernatants were then removed and the solvent evaporated in an oven. The dry residue containing the starch was incubated with sodium hydroxide (0.5 N) at 60°C for 1 h, neutralized with hydrochloric acid (0.5 N), treated with amylase (amyloglucosidase, Sigma–Aldrich, USA) dissolved in citrate buffer (pH 4.6) to break the starch down to glucose and incubated for 30 min at 60°C. The supernatants were then mixed with NADP/ATP and HK/G6P-DH-solution (Enzytec^TM^ E1210, r-biopharm, Germany) and the absorption of NADPH (dependent on starch content) measured with a UV/Vis spectrophotometer (Lambda 20, Perkin Elmer, USA) at 340 nm before and after the addition of the latter solution. Starch content (SC; %) was calculated as:

SC =c×100×V/m

where *c* is the starch concentration (g/L) in the measured solution, *m* is the mass of plant material (g) and *V* (L) is the volume of the solution in the spectrophotometer (also see Mayr et al., 2014).

### Shoot Phenology and Length of White Roots

The phenology of the shoot was monitored from winter to the initial stages of fruit development. Phenological stages were classified according to the BBCH-scale (Chapman and Catlin, 1976; Meier, 2003; see **Table 1**). To investigate xylem development, cross-sections were made with a microtome and current year ring width was measured on five shoots per treatment. In 2013, after removal of the insulation, 8–10 roots from three trees per treatment were harvested at 10 cm depth (the main root zone) and taken to the laboratory. The length of each visible white root was analyzed with a stereo microscope (Olympus SZ61, Olympus Austria, Vienna, Austria) interfaced with a digital camera (Sony Cyber-shot DSC-W17) and image analysis software [ImageJ, 1.37, National Institutes of Health (NIH), Bethesda, MD, USA, public domain].

**Table 1:** Phenological stages of apple trees according to the BBCH-scale (Chapman and Catlin, 1976; Meier, 2003).

BBCH-code	Stage and description
00	Winter bud
	*Winter dormancy; buds closed and covered by dark brown scales*
51–52	Silver tip (bud swelling)
	*Visibly swollen buds, bud scales elongated with light colored patches, often covered by hairs*
53	Green tip (bud break)
	*Bud break, first green leaf tips visible*
54	½ inch green
	>*10 mm of leaf tissue is projecting from the buds*
56	Tight cluster
	*Blossom buds mostly exposed but still closed, tightly grouped*
57	First pink
	*Sepals slightly open, petals visible (pink)*
59	Full pink
	*Most blossoms balloon-shaped*
61	First bloom
	*About 10% of blossom buds open*
65	Full bloom
	*At least 50% of blossom buds open, fall of first petals*
69	Post bloom
	*All petals fallen*
71	Fruit < 10 mm
72	Fruit < 20 mm


### Statistics

Differences between treatments in the levels of native embolism, water and starch contents and lengths of white roots were tested with the Student’s *t*-test (normal distribution and equal variances) or the non-parametric Mann–Whitney *U* Test (no normal distribution and/or unequal variances). All tests were made pairwise at a probability level of 5% using SPSS version 21. Correlation coefficients were tested with the Pearson’s product-moment coefficient.

## Results

### Weather Conditions and Insulation

**FIGURE 2:**
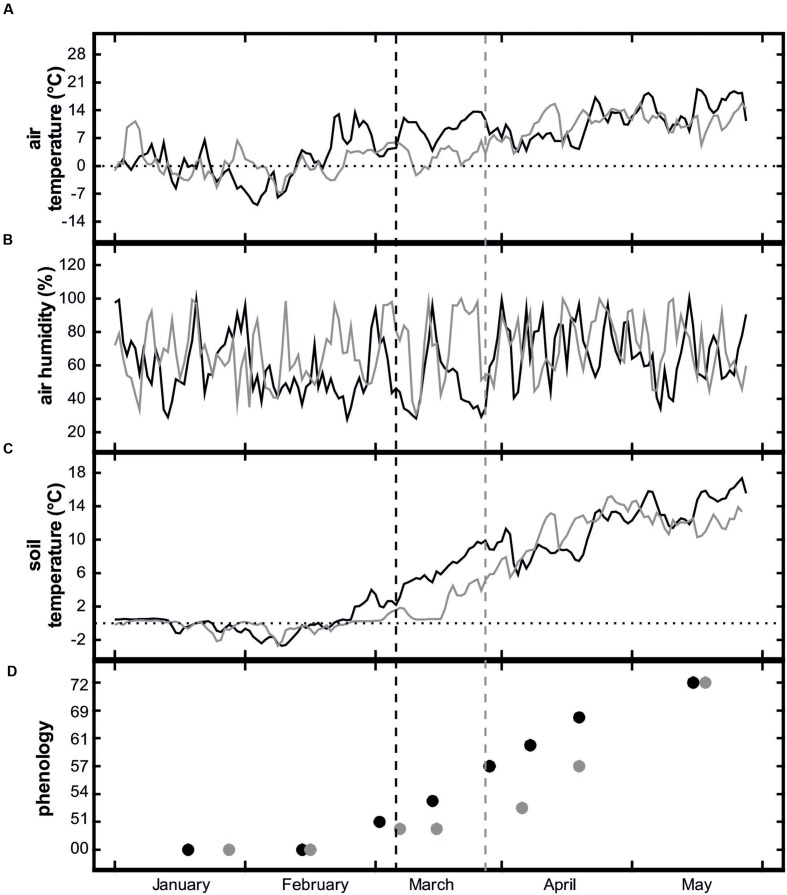
**Mean daily air temperature **(A)**, air humidity **(B)**, soil temperature at 10 cm depth **(C)**, and phenological stages of *Malus domestica* ‘Golden Delicious’ **(D)** of control plots from January to June 2012 (black lines and symbols) and 2013 (gray lines and symbols).** Dashed vertical lines show bud break. For an explanation of the phenological stages, see **Table 1**.

Weather conditions differed considerably between the 2 years of the study. The following description is based on the monthly climate report of the South Tyrolean Weather Service as well as our own measurements with the meteorological station in the orchard. The first half of February 2012 was exceptionally cold with mean air temperatures about 1.5°C below the long-term average. The cold period was followed by a sudden increase in temperature and extraordinarily warm temperatures in the second half of February (**Figure 2A**). This caused an early onset of growth and one of the earliest bud breaks ever observed in the study area (South Tyrolean Advisory Service for Fruit and Wine-growing, pers. comm.). Besides temperature extremes, February 2012 was also extremely dry due to low precipitation and foehn winds. Also, March 2012 was exceptionally warm with mean air temperatures about 3.2°C above the long-term average. In April, air temperatures were close to the long-term average and precipitation was significantly higher.

**FIGURE 3:**
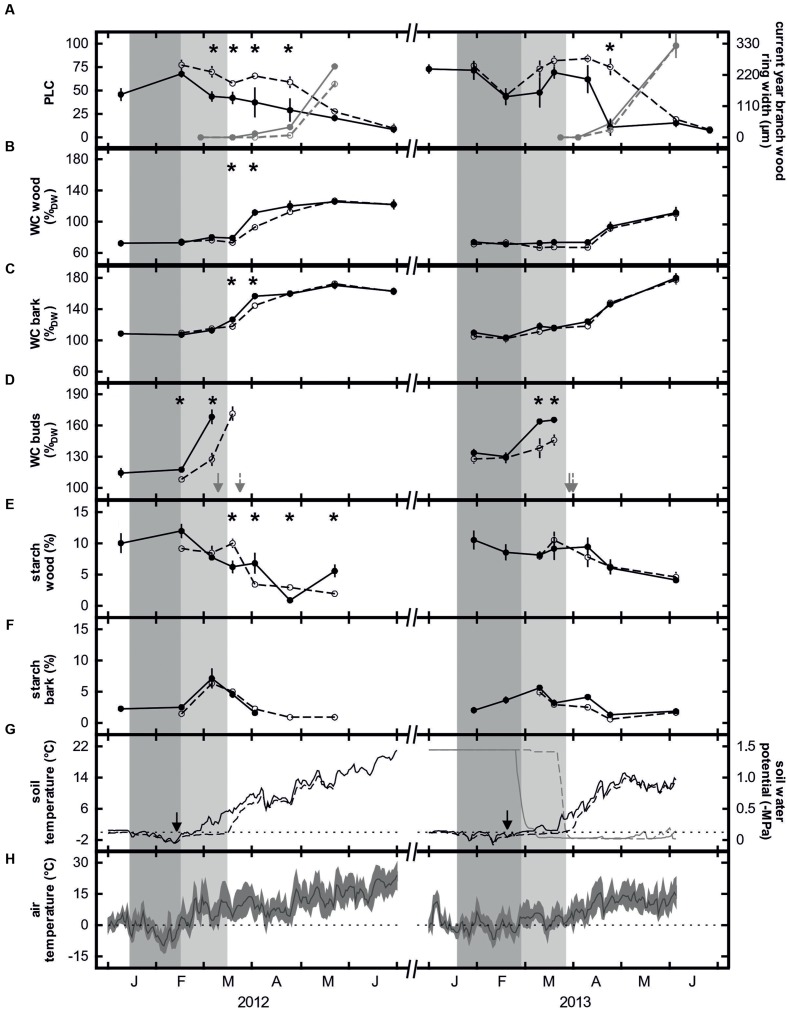
**Means and standard errors of percentage loss of hydraulic conductance (black) and current year branch wood ring width (gray; **A**), water contents of wood **(B)**, bark **(C)**, and buds **(D)**, starch contents of wood **(E)** and bark **(F)**, daily mean soil temperature (black lines) and soil water potential (gray lines) at 10 cm depth **(G)** and daily mean (line), maximum and minimum (area) air temperature **(H)** of control trees (closed symbols, solid lines) and trees subjected to prolonged soil frost (open symbols, dashed lines) in spring 2012 and 2013.** Arrows in panel **(D)** show the bud break of the control (solid) and treated (dashed) trees and in panel **(G)** the installation of the insulation plates. Dotted horizontal lines indicate 0°C **(G,H)**. Dark gray bars indicate sub-zero soil temperatures in control plots and light gray bars the period of prolonged soil frost in treated plots. Asterisks indicate significant differences between control and treated trees. Please note that soil water potential was only measured in 2013 (*n* = 5).

January 2013 also experienced above-average air temperatures and precipitation but (in contrast to 2012) cold periods occurred in February and March. Although relatively mild temperatures at the end of February and the beginning of March induced the onset of bud swelling, the low temperatures in the second half of March stopped further development until temperatures again increased at the end of March and beginning of April (**Figure 2D**). Because of the air temperature differences between 2012 and 2013, soil temperatures also differed considerably. In 2012, the soil in the control plots thawed during the second half of February and by mid-March, mean soil temperatures were consistently above 4°C (**Figure 2C**). In 2013, soil thawing also started at the end of February but soil temperatures remained low until the end of March. Due to the early irrigation start in February, in both study years soil water potential never fell below -0.30 MPa from soil thawing in January/February to freezing in November/December (**Figure 3G**, also see Beikircher et al., 2013).

Insulation of the soil with the Styrofoam sheets prolonged soil frosting for several weeks. In 2012, daily mean soil temperatures in the control plots rose above zero on 23 February, while that in the insulated plots remained frozen for another 23 days. Differences in soil temperature between plots ranged from about 1 to 6°C, with a difference greater than 3°C on 11 days (**Figure 3G**). In contrast, in 2013, soil thawing started about 4 weeks earlier in the control plots (27th February) compared with the insulated plots. However, due to the subsequent weather conditions, temperature differences were small. Differences were less than 1°C on 17 days and greater than 3°C only on the last 2 days of insulation (**Figure 3G**). We cannot completely exclude that the insulation has altered soil water relations by preventing precipitation from reaching the root zone. However, as insulation was immediately removed when soil started thawing (see “Materials and Methods” section) and due to the daily irrigation from February onward, soil water availability upon thawing was high for both control and treated trees. Accordingly, soil water potential measurements in 2013 revealed similar values upon natural thawing and removal of insulation, respectively (**Figure 3G**).

### Native Embolism, Water, and Starch Content

**FIGURE 4:**
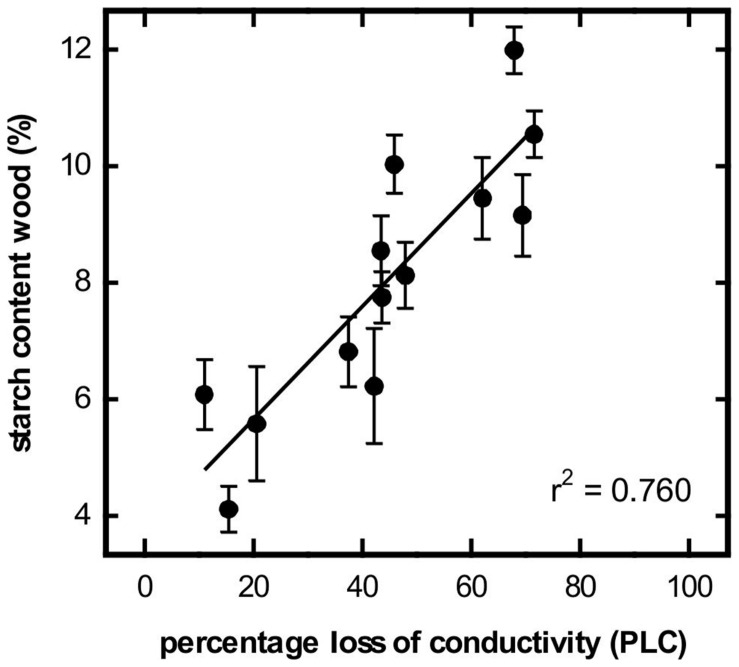
**Relationship between starch content of wood (%) and percentage loss of hydraulic conductivity (PLC).** Values are from control trees in spring 2012 and 2013 (*P* = 0.05).

In January of both years of the study, a hydraulic conductance loss of about 70% was measured in the control trees. Due to refilling processes, the level of native embolism decreased to less than 10% by the end of spring (**Figure 3A**). Simultaneously, the water contents of wood, bark and buds increased strongly (**Figures 3B–D**). Prolonging the soil frost by surface insulation had a strong influence on embolism reversal, particularly in 2012. Significant differences between control and insulated plots were found from 6 March to 24 April. In contrast, in 2013, significant differences were found only on one sampling date (24 April; **Figure 3A**). In 2012, simultaneous increases in the water contents of wood, bark and buds occurred in both treatments but were significantly higher in the control trees. In contrast, embolism differences had already leveled off by April 2012. In March 2013, the water contents of wood and buds were slightly higher in the control trees but significant differences were only found for the buds (**Figures 3B–D**). In contrast, midday water potential from bud break (after removal of insulation) to June was relatively constant and ranged between -0.23 and -1.45 MPa. No differences between treatments were observed. Starch contents in the bark were low during winter (ca. 2%), increased around bud break (7%) but then decreased again during refilling. No significant differences between control and treated trees were observed (**Figure 3F**). In contrast, xylem starch content was consistently high during winter (9–11%). In 2012, it decreased to almost 0% in the control trees and to about 2% in the treated trees with the differences being significant from mid-March to the end of May. In 2013, the starch content of the wood decreased to only about 4%, with treatment differences being non-significant (**Figure 3E**). In control trees, for both study years a strong correlation between PLC and starch content of the xylem was found (**Figure 4**).

### Phenology and Length of White Roots

**FIGURE 5:**
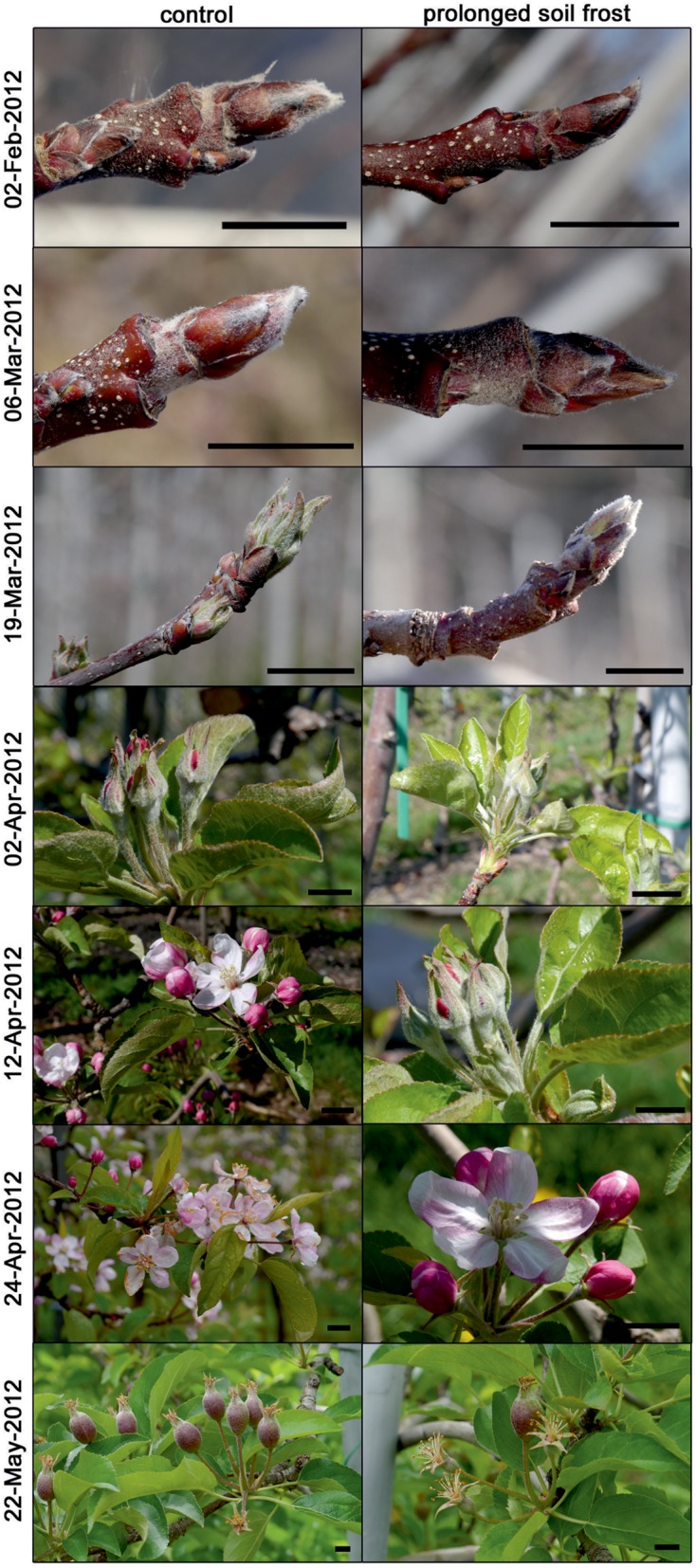
**Typical phenological stages of control and treated trees of *M. domestica* from February to May 2012.** Bars 1 cm.

In 2012, bud break occurred about 3 weeks earlier than in 2013 (**Figures 2D** and **3D**) with prolonged soil frost significantly affecting phenology. Bud break of treated trees was about 9 days after that of the control trees and the subsequent phenological stages were also delayed in the treated trees, compared to the controls (**Figure 5**). In 2013, there were no significant phenological differences between treatments (data not shown).

**FIGURE 6:**
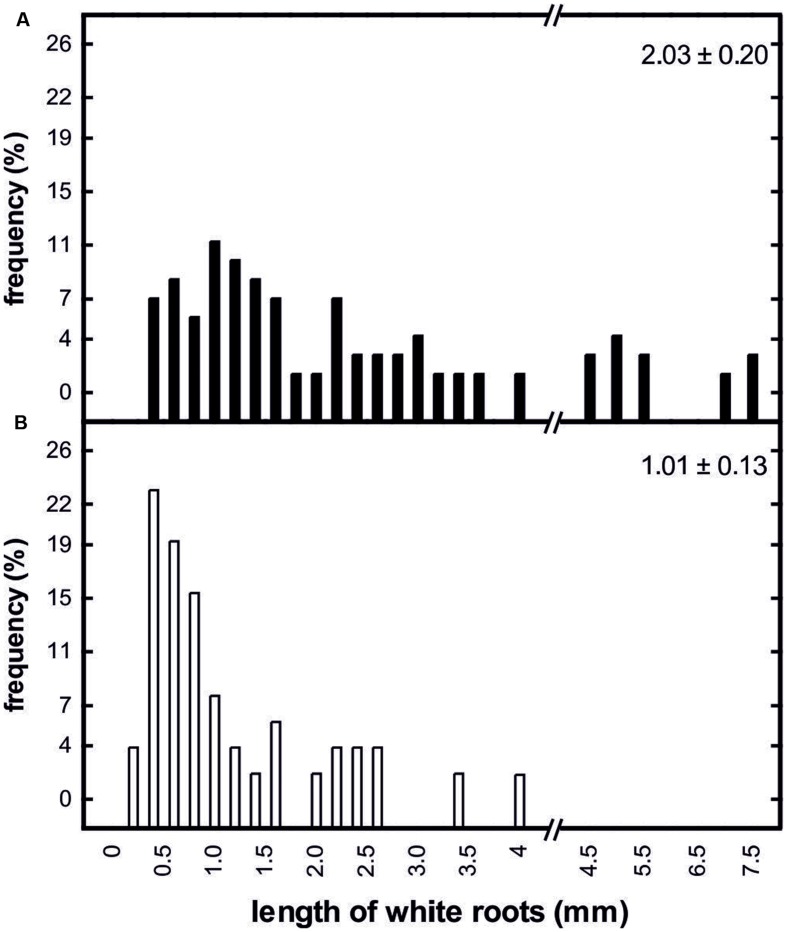
**Frequency distribution of the length of white roots from control trees (**A**; *n* = 70) and trees subjected to prolonged soil frost (**B**; *n* = 50) on April 10, 2013.** The relative numbers of white roots is given for 0.2 mm (left site) and 0.5 mm (right site) classes, respectively. Values on the upper right of the panels show means and standard errors of the length of white roots on the sampling day.

Root growth was clearly affected by prolonged soil frost. On 24 April 2013, about 10 days after bud break in control trees, the mean length of white roots of control trees (2.03 ± 0.20 mm) was significantly higher than that of treated trees (1.02 ± 0.13 mm; **Figure 6**). In contrast to the treated trees, the control trees had developed numerous white roots with lengths greater than 4 mm (**Figure 6**).

## Discussion

During the vegetation period, irrigation management in most commercial apple orchards is optimized to ensure high quality and productivity (Naor and Girona, 2012; also see Beikircher et al., 2013). However, hydraulic limitations of growth and yield are related not only to summer drought. During autumn and winter, the soil–plant-atmosphere continuum is interrupted by winter embolism and inhibited water uptake from the frozen soil (frost drought). Transpirational water loss by the tree due to late or failed leaf shedding or from the periderm can further lower the water status of apple trees in winter (Beikircher and Mayr, 2013). A timely restoration of the tree’s hydraulic transport system (stem) and an efficient re-connection to the soil water (roots) in spring is thus crucial. In this study we analyzed the impacts of prolonged soil frost on xylem recovery and growth in *M. domestica* var. ‘Golden Delicious.’

### Xylem Recovery and Phenology of Control Trees

Weather conditions varied considerably between the two study years. While the spring of 2012 was exceptionally warm and dry, the weather in 2013 was rather cold and wet. This had strong influences on the time course of the trees’ xylem recovery and growth. In mid-winter of both years, the loss of hydraulic conductance in control trees was about 70% but this decreased to negligible values in spring (**Figure 3A**). A similar pattern of winter embolism and subsequent xylem recovery has been reported for many angiosperm species (e.g., Sperry et al., 1988a, 1994; Sperry and Sullivan, 1992; Cochard et al., 1997; Ameglio et al., 2002) inclusive *M. domestica* var. ‘Alberta green’ (Christensen-Dalsgaard and Tyree, 2014). In February 2012, a rapid increase in air and soil temperatures triggered the restoration of the hydraulic conductance in the stem with the level of native embolism decreasing steadily until May. In contrast, air and soil temperatures in 2013 remained below 5°C until the end of March. In consequence, the level of embolism remained high (about 60%) until April when it decreased rapidly as soon as weather conditions became more favorable (**Figure 3**). However, in both years the level of native embolism was below 10% by June. This observation is similar to that of Christensen-Dalsgaard and Tyree (2014), who also found only small differences in the refilling success of apple, willow and poplar trees, despite significantly different weather conditions during spring. The residual 10% loss of hydraulic conductance may be due to damage, cavitation- or frost-fatigue of conduits (Hacke et al., 2001; Christensen-Dalsgaard and Tyree, 2014).

In 2012, a significant decrease in native embolism was observed prior to the growth of new conduits (level of native embolism in February differed significantly from following values; significance not shown in **Figure 3**), while in 2013 both processes occurred in parallel. Thus, in *M. domestica*, xylem recovery is a result not only of the differentiation of new xylem conduits but also of the refilling of embolized ones (also see Christensen-Dalsgaard and Tyree, 2014).

Refilling of embolized conduits in spring can be related to positive xylem pressures. The genus *Malus* has been reported to develop root pressure in spring but to a much lesser extent than maple, walnut or birch for instance (Wiegand, 1906). Thus, root pressure might have been involved in very first refilling processes. However, in *Malus* as in other species, root pressure ceases before or around bud break (e.g., Wiegand, 1906; Sperry et al., 1994; Cochard et al., 2001; Ewers et al., 2001; Ameglio et al., 2002; Hao et al., 2013). At that time, refilling is not yet completed for several more weeks (Sperry et al., 1994; Christensen-Dalsgaard and Tyree, 2014). Recent studies indicate that vessel refilling in the absence of positive pressures is based on starch depolymerisation and subsequent water inflow to embolized conduits (see ‘Introduction’). However, to our knowledge, there is only one other study linking xylem recovery in spring with seasonal patterns in starch content: In their study, Mayr et al. (2014) found a close relationship between starch content and refilling in *Picea abies*. In our study trees, vessel refilling coincided with a decrease in the starch contents of both bark and wood (**Figure 3**). Thereby, decrease in native embolism was strongly correlated with the decrease of starch in the wood (**Figure 4**). In the bark, prior to the decrease in spring, an increase in starch content was observed in late winter, which is a commonly observed phenomenon in many temperate tree species (Essiamah and Eschrich, 1985; Sauter and van Cleve, 1994; Ameglio et al., 2004). Contemporary with the formation of new conduits (increase in current year branch wood ring width), a significant increase in wood water content was observed (**Figure 3**). Strati et al. (2003) and Hao et al. (2013) found the increase in the wood’s volumetric water content was correlated with the refilling of embolised conduits. The increase in the water contents of the bark and buds started earlier than of the wood and was likely related to water uptake by young cells as they extended and grew (Larcher, 2003; Neuner and Beikircher, 2010).

Weather conditions during spring influenced not only tree hydraulics but they also had significant effects on tree phenology. In February 2012, a strong increase in bud water content was observed, followed by an exceptionally early bud break on March 10th (**Figure 3D**). Obviously, this was related to favorable weather conditions lasting for several weeks. In 2013, bud swelling also started in February but due to the subsequent unfavorable weather conditions, the buds remained in this phenological stage until the beginning of April (**Figures 2** and **3**).

### Impact of Prolonged Soil Frost on Hydraulics and Phenology

Prolongation of soil frost for 3–4 weeks clearly affected apple tree hydraulics and phenology. The greatest effects were observed in 2012, when soil temperatures differed considerably between control and treated trees (**Figure 3G**). About 14 days after the first significant decrease in embolism of control trees (February), a significant but small decrease was also observed in the treated trees. However, the level of native embolism in treated trees then remained stable at around 60%. Despite starch depolymerisation in the wood at the beginning of April, the next significant decrease in embolism was not observed until the end of April. This indicates that limited uptake of water from the soil, and not starch metabolism, was the determining factor for xylem recovery in the treated trees. This hypothesis can be further proofed by the two-phase relationship between soil temperature and level of native embolism: Up to about 7°C level of embolism remained constant and then decreases rapidly toward 0% embolism (data not shown). Studies on Scots pine in the boreal region also showed a delay in the onset of sap flow when soil thawing lagged behind the start of the growing season (Repo et al., 2008). As with recovery from embolism, the water contents of wood, bark and buds showed a delayed increase in the treated trees, even though differences had already leveled off at the beginning of April (**Figures 3B–D**). In contrast to 2012, differences in the soil temperatures of control and treated trees were less dramatic in spring 2013. Due to cold and wet weather conditions, the soil in the control plots was only slightly above freezing until the end of March. As a consequence, the loss of hydraulic conductance remained high in all trees and significant differences were found only at the end of April (**Figure 3A**). This shows that not only soil frost but also cold soils slow xylem recovery in apple trees. However, in contrast to Repo et al. (2005) who found that a 2-week delay in soil thawing led to the death of Scots pine seedlings, we did not observe any visible damage of treated trees.

Frozen or cold soils strongly limit water uptake due to reduced root water permeability, increased transfer resistances, and inhibited fine root growth (see ‘Introduction’). Water uptake in trees occurs mainly in the fine roots (Pallardy, 2008). Newly formed roots are white, unsuberized and highly permeable to water. In apple trees, white roots live for from 1 to 4 weeks in summer and for up to 3 months in winter (Wells and Eissenstat, 2001; Pallardy, 2008). Therefore, fine root survival rate during winter depends strongly on the prevailing soil conditions (Psarras et al., 2000; Wells and Eissenstat, 2001; Pallardy, 2008). According to Rogers (1939), the onset of fine root growth in apple trees starts at soil temperatures above 6°C and also according to Alvarez-Uria and Körner (2007), 6°C is a critical temperature limit for root growth in many temperate trees. Several authors reported that the main period of root growth starts several weeks after bud break (Head, 1967; Psarras et al., 2000; Wells and Eissenstat, 2001). We analyzed the length of white roots 10 days after bud break on April 10th, 2013. At this stage, the soil temperature around the control trees lay between 4 and 6°C for about 2 weeks but for only a few days around the treated trees. This difference had a strong effect: the white-root length of the control trees was about twice that of the treated trees (**Figure 6**). Although water uptake in spring may occur partly via overwintering fine roots and older roots (Landsberg and Jones, 1981; Wells and Eissenstat, 2001, 2003), it is likely that the observed limitation in fine root growth negatively influenced xylem recovery. However, further research is required to proof this hypothesis and determine the importance of water uptake by white roots for refilling.

Besides xylem recovery, prolonged soil frost also had strong impacts on phenology. In 2012, bud break occurred about 10 days later in treated trees than in control trees. As a consequence, subsequent phenological stages were also delayed but the differences diminished with time and were negligible by June (**Figure 5**). In 2013, a short, warm spell at the beginning of March triggered bud swelling (also evidenced by increased bud water content) in control trees but due to the later deterioration in weather, the trees remained at this phenological stage. Thus, bud break was almost contemporary in control and treated trees. Greer et al. (2006) also found bud break to be strongly correlated with soil temperatures. According to these authors, apple trees store carbohydrates predominately in the roots, and low soil temperatures affect bud break by reducing carbohydrate remobilisation. However, growth also strongly depends on water status. While water stored within the tree may be sufficient for the very earliest growth processes, further development requires access to soil water.

Overall, it is evident that prolonged soil frost critically affects both tree hydraulics and phenology but these are affected to different extents. While for the level of embolism, significant differences between treatments were observed from the beginning of March to the end of April 2012, growth-related parameters showed less pronounced differences. For instance, bud break was delayed by only 10 days in 2012. Though water availability is undoubtedly important for growth, air temperatures also play a major role.

## Conclusion

Successful restoration of the hydraulic system after winter is essential for the survival of temperate trees. Nevertheless, there are remarkably few studies dealing with the recovery of the hydraulic system in spring, its underlying mechanisms and possible limitations. There are some studies focusing on seasonal patterns in xylem embolism, some dealing with the impact of prolonged soil frost on sap flow and some on fine root growth in spring (see above). Many of those studies have been carried out on conifers. To our knowledge, our study is the first (I) showing the impact of prolonged soil frost on xylem recovery and phenology by (II) correlating xylem functionality to starch content and xylogenetic aspects and (III) including fine root growth in an angiosperm over (IV) two consecutive spring seasons with contrasting weather conditions.

Our findings show that prolonged soil frost can have strong impacts on xylem recovery and phenology of apple trees in spring. The main reason therefore is the limited water uptake from the cold soil due to increased resistance and impaired fine root growth rather than starch metabolism. However, to which extent prolonged soil frost affects xylem recovery and tree phenology strongly depends on weather conditions. Thus, prolonged soil frost may be critical when a number of unfavorable conditions coincide. For example, when soil frost persists in conjunction with early bud break and thus high water demand for transpiration and growth, or weather conditions favoring high transpirational demand, or increased water loss from the periderm of lammas shoots in winter and spring (see Beikircher and Mayr, 2013), or reduced water storage capacity. Other factors limiting water uptake are cold soil and high fine-root mortality due to earlier frost injury (Groffman et al., 2001; Tierney et al., 2001). These may lead to the severe winter damage as observed at irregular intervals in the apple orchards of northern Italy (see ‘Introduction’). In particular locations, where some of these factors occur fairly often, apple production may not be commercially sustainable. However, facing climate change prolonged soils frost may also have implications for other trees and other ecosystems such as boreal forests when soil thawing lags behind the start of the growing season.

## Author Contributions

BB organized the field experiment, carried out the measurements in the second study year, supervised the measurements of CM in the first study year and wrote the presented manuscript. In the frame of her master thesis, CM carried out all measurements of the first study year. SM is leader of the research group, wrote the project proposal financing the presented study, supervised the experiment, counseled the PostDoc BB and revised the presented manuscript.

## Conflict of Interest Statement

The authors declare that the research was conducted in the absence of any commercial or financial relationships that could be construed as a potential conflict of interest.
